# Method-Dependent Variability in Hempseed Lipidomics: Combined Influence of Extraction, Derivatisation, and Genotype on Fatty Acid Composition and Stability

**DOI:** 10.3390/molecules31142480

**Published:** 2026-07-16

**Authors:** Doris Floares (Oarga), Iuliana Popescu, Diana Obistioiu, Anca Hulea, Adina Berbecea, Isidora Radulov

**Affiliations:** Faculty of Agriculture, University of Life Sciences “King Mihai I” from Timisoara, Calea Aradului 119, 300645 Timisoara, Romania; doris.oarga@usvt.ro (D.F.); dianaobistioiu@usvt.ro (D.O.); anca.hulea@usvt.ro (A.H.); adina_berbecea@usvt.ro (A.B.); isidora_radulov@usvt.ro (I.R.)

**Keywords:** *Cannabis sativa* L., fatty acid composition, microwave extraction, oxidative stability

## Abstract

Hempseed (*Cannabis sativa* L.) is recognised as a valuable source of nutritionally important lipids, particularly polyunsaturated fatty acids (PUFA). Despite growing interest in hempseed oil, the combined influence of extraction and derivatisation on fatty acid profiling remains poorly understood, limiting comparability and standardisation of lipidomic analyses, particularly for fibre-type hemp cultivars. This study evaluated the effects of microwave-assisted and Soxhlet extraction, combined with acidic and basic derivatisation, on fatty acid composition, lipid quality indices, and oxidative stability in Romanian fibre-type hempseed cultivars. The analysed oils were dominated by linoleic and α-linolenic acids, with favourable PUFA/SFA and ω-6/ω-3 ratios, low atherogenic and thrombogenic indices, and high hypocholesterolaemic/hypercholesterolaemic ratios, indicating excellent nutritional quality. Statistical analyses confirmed that extraction and derivatisation significantly influenced fatty acid composition and lipid quality indices, while principal component analysis identified extraction methodology as the main source of variability. Oxidative stability showed moderate correlations with fatty acid composition, suggesting that differences in lipid profiles may influence susceptibility to oxidation. Overall, the study demonstrates that Romanian fibre-type hemp cultivars are valuable sources of nutritionally favourable lipids and emphasises the need for standardised extraction and derivatisation protocols to improve the reliability and comparability of hempseed lipidomic analyses.

## 1. Introduction

Fatty acids (FA) are fundamental components of biological systems, having essential roles in the structure of cell membranes, the regulation of enzymatic activity and the modulation of inflammatory processes [1]. From a chemical point of view, they are long-chain hydrocarbon carboxylic acids, classified according to the degree of unsaturation into saturated fatty acids (SFA), monounsaturated fatty acids (MUFA) and polyunsaturated fatty acids (PUFA) [2]. Among PUFAs, α-linolenic acid (ALA, ω-3) and linoleic acid (LA, ω-6) are considered essential because the human body cannot synthesise them and must obtain them through diet [3].

ω-3 and ω-6 PUFA play critical biological roles from the early stages of development, contributing to the organisation of cell membranes, energy metabolism, intracellular signal transmission and the regulation of gene expression [4]. An adequate intake of essential fatty acids is associated with a reduced risk of cardiovascular disease [5,6], metabolic disorders associated with obesity [7] and degenerative diseases [8]. In addition, linoleic acid has an essential structural role in maintaining the skin barrier function, being a major component of epidermal ceramides [9].

**Figure 1:**
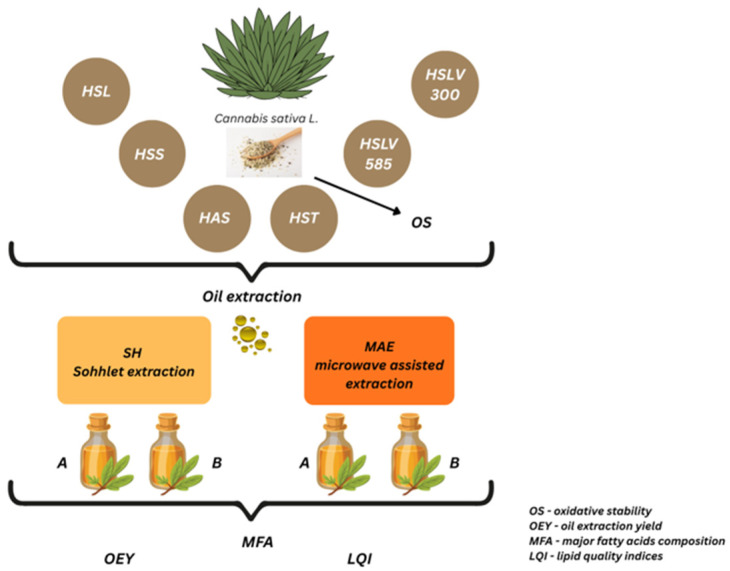
Integrated effects of extraction method and genotype on hempseed lipidomic profiles.

From a nutritional perspective, not only the quantity but also the distribution of different classes of fatty acids is important. The optimal ω-6/ω-3 ratio is considered to be between 1:1 and 4:1 [10]. The evaluation of lipid quality is frequently performed through indices such as the atherogenic index (IA), the thrombogenic index (TI), and the hypocholesterolaemic/hypercholesterolaemic (h/H) ratio [11]. These parameters allow the assessment of the potential impact of dietary lipids on cardiovascular and metabolic health. In this context, vegetable oils are major sources of essential fatty acids and bioactive compounds, and oilseed crops have become of interest both for the food industry and for biomedical research [12,13]. Among them, hemp seeds (*Cannabis sativa* L.) have attracted interest due to their high lipid content and favourable fatty acid profile. Hemp oil contains over 90% unsaturated fatty acids and has an ω-6/ω-3 ratio close to recommended values for human nutrition [14]. Also, the low content of saturated fatty acids and the high level of PUFA give this oil a nutritional profile associated with beneficial effects on cardiovascular health [15]. Recent studies have provided detailed insights into the fatty acid composition of *Cannabis sativa* seeds, highlighting the predominance of polyunsaturated fatty acids and the variability associated with cultivar and processing conditions. Rosso et al. reported that hemp seeds contain 71.43–74.25% polyunsaturated fatty acids, with linoleic acid representing 53.17–54.86% of total fatty acids and α-linolenic acid accounting for 17.56–18.44%, emphasising the high nutritional value of hemp lipids [16]. In a comprehensive evaluation of 29 industrial hemp cultivars, Arango et al. also identified linoleic acid as the major fatty acid, comprising more than half of the total fatty acid content, while significant genotype-dependent differences were observed for α-linolenic, oleic, palmitic, and γ-linolenic acids [17]. Likewise, Alonso-Esteban et al. investigated the fatty acid composition of eight unhusked hemp seed varieties and eight commercial hulled hemp seed samples, reporting linoleic acid as the predominant fatty acid in both seed types, with average concentrations of 16.75 g/100 g in unhusked seeds and 26.10–29.74 g/100 g in hulled seeds. The content of α-linolenic acid ranged from 4.25 g/100 g in unhusked seeds to 7.82–9.78 g/100 g in hulled seeds, whereas oleic acid averaged 4.10 g/100 g and 5.77–7.43 g/100 g, respectively, demonstrating differences associated with seed processing and variety [18]. More recently, Allay et al. optimised the microwave-assisted extraction of hemp seed oil and reported a lipid fraction composed of 65.30% polyunsaturated fatty acids, 18.41% monounsaturated fatty acids, and 15.10% saturated fatty acids, with linoleic acid (50.23%), oleic acid (18.23%), α-linolenic acid (14.09%), palmitic acid (10.30%), and stearic acid (3.09%) identified as the major constituents [19]. Collectively, these studies demonstrate that the lipid composition of hemp seeds is influenced by both biological factors (genotype, soil conditions, and maturity) and the analytical methodology used for the extraction and determination of fatty acids. Figure 1 illustrates the combined influence of extraction method and genotype on hempseed lipidomic profiles, highlighting the importance of extraction technique selection for preserving native lipid compounds and ensuring reproducible analytical results, with Soxhlet extraction remaining the conventional reference method due to its high efficiency despite its substantial solvent consumption and lengthy processing time [20]. In contrast, microwave-assisted extraction (MV) offers advantages such as reduced extraction time, reduced solvent consumption and increased recovery yield of bioactive compounds [19].

Similarly, the derivatisation step significantly influences the chromatographic analysis of fatty acids. Since free fatty acids are not volatile enough for GC-MS analysis, they are converted to fatty acid methyl esters (FAME) by acid or base derivatisation [21,22]. However, different derivatisation protocols can selectively affect the recovery of specific lipid classes, particularly oxidation-sensitive PUFAs, leading to significant variations in reported lipid profiles.

Although numerous studies have investigated the composition of hemp oil, most have focused on commercial or oleaginous varieties and have evaluated the effects of extraction or derivatisation separately. The combined influence of extraction technique and derivatisation protocol on fatty acid composition, lipid quality indices, and oxidative stability remains underexplored, especially in the case of fibrous hemp varieties. Furthermore, the interaction between the biological variability of genotypes and the variability introduced by the analytical methodology is a major source of inconsistency across studies and a challenge to the standardisation of lipidomic analyses.

In this context, the Romanian fibrous hemp varieties developed at the Lovrin Agricultural Research and Development Station provide a valuable biological model for investigating lipid variability. These genotypes, primarily developed for industrial applications, are insufficiently characterised from a nutritional perspective, although they may represent important alternative sources of PUFA-rich oils and bioactive compounds.

Therefore, the present study aims to systematically evaluate the individual and interactive effects of the extraction method (Soxhlet versus microwave-assisted extraction), the derivatisation protocol (acid versus basic) and the genotype on the fatty acid composition, lipid quality indices and oxidative stability of hemp seeds. Multivariate statistical analyses, including three-way ANOVA and principal component analysis (PCA), were applied to elucidate the relative contributions of biological and methodological factors. To our knowledge, this is the first study to simultaneously investigate the extraction–derivatisation–genotype interactions on the lipid profile of fibrous hemp seeds. At the same time, the study represents the first comprehensive multivariate evaluation of Romanian fibrous hemp varieties. It provides methodological information relevant for the standardisation of lipidomic analyses and for improving the comparability of data on the lipid composition of hemp seeds.

## 2. Results and Discussions

### 2.1. Oil Extraction Yield by Microwave and Soxhlet Methods

**Figure 2:**
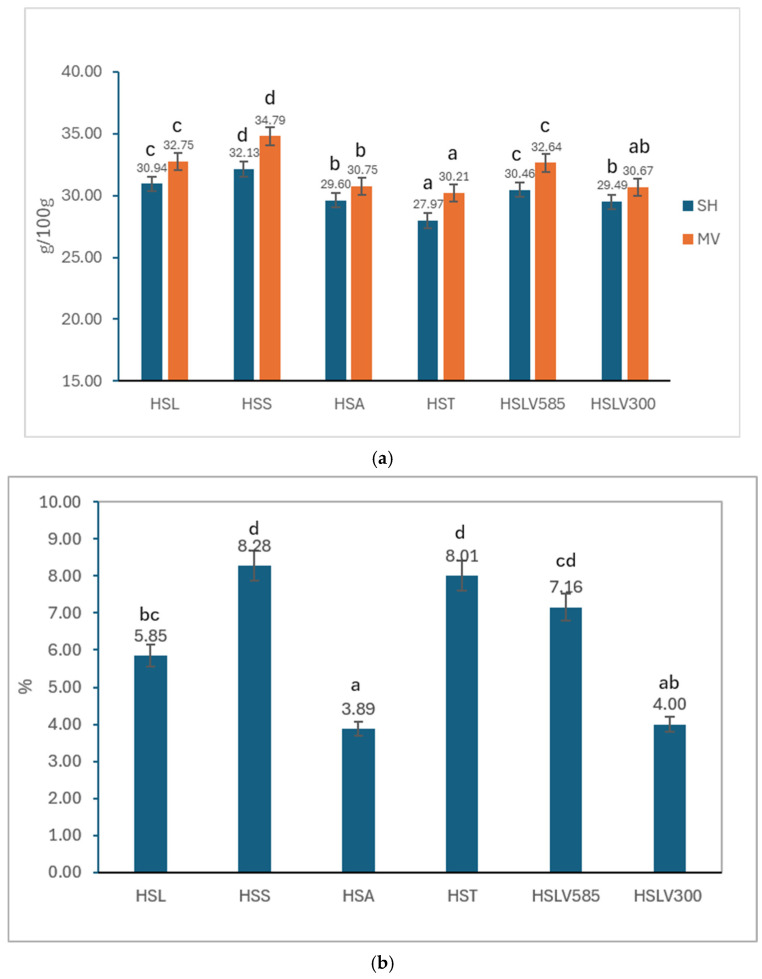
(**a**) Oil content of samples extracted using the SH and MV methods. Values are expressed as mean ± standard deviation (SD) (*n* = 3). Based on the ANOVA results followed by Tukey’s post hoc test, different lowercase letters (a–d) indicate significant differences (*p* < 0.05) between samples obtained with the same extraction method. Hempseed samples: HSL—Lovrin 110, HSS—Silvana, HSA—Armanca, HST—Teodora, HSLV585—Lovrin 585, HSLV300—Lovrin 300, MV—microwave extraction; SH—Soxhlet extraction. (**b**) Increase in oil yield (%) using microwave and Soxhlet extraction methods. Values are presented as mean ± standard deviation (SD) (*n* = 3). Based on one-way ANOVA with Tukey’s post hoc test, different lowercase letters (a–d) indicate significant differences (*p* < 0.05). Hempseed sample: HSL—Lovrin 110, HSS—Silvana, HSA—Armanca, HST—Teodora, HSLV585—Lovrin 585, HSLV300—Lovrin 300.

The oil content of hempseed samples extracted by Soxhlet (SH) and microwave-assisted extraction (MV) are presented in Figure 2.

Figure 2a illustrates the total lipid content (g/100 g), whereas Figure 2b shows the relative increase in extraction yield obtained by MV compared with SH and calculated according to Equation (2).

According to Figure 2, oil contents determined by SH ranged from 27.97 to 32.13 g/100 g, whereas MV ranged from 30.21 to 34.79 g/100 g. In the SH dataset, no statistically significant differences were observed between HSA and HSLV300, or between HSL and HSLV585 (*p* > 0.05). Under the experimental conditions used, the higher oil recoveries observed with MV may reflect the combined effects of microwave heating, extraction conditions, and solvent availability. Similar effects have been described in previous studies on hempseed and other oilseed matrices, where microwave irradiation promoted rapid volumetric heating and influenced extraction relative to conventional solvent extraction methods [19,23,24]. The most significant increase under MV was for HSS (8.28%), while HSA showed the smallest increase (3.89%). The lipid contents obtained for the analysed hempseed cultivars are consistent with values previously reported in the literature. Rosso et al. examined three cultivars—Carmaenecta, Enectaliana, and Enectarol—grown in central Italy to evaluate their lipid composition and antioxidant potential for monogastric feeds; after HCl acid hydrolysis, lipids were extracted using Soxhlet, resulting in total lipid contents of 30.14–30.93 g/100 g of the sample [16]. In another study, Ma et al. reported 28.50 ± 5.60 wt% lipids in Calgary-grown hempseed extracted with petroleum ether in a Soxhlet extractor [25]. Similar values have been reported in the literature, including 24.50% by Siano et al. [26]; 25.40–33.00 g/100 g, as reported by House et al. [27]; and 26.90–30.60 g/100 g as determined by ether extract in the work of Vonapartis et al. [28]. Using initial acid hydrolysis followed by diethyl ether extraction, Pihlanto et al. measured total lipids at 23.10 g/100 g, which is lower than the value found in our study [29].

Microwave-assisted extraction is increasingly recognised as a process-intensified, energy-efficient, and easily automated alternative to conventional solvent extraction. Its advantages stem from the ability of microwave irradiation to enhance mass transfer, accelerate solvent penetration into plant tissues, and promote rapid cell disruption, thereby improving lipid recovery while substantially reducing extraction time. These mechanistic benefits are well-documented across food, biomedical, and pharmaceutical applications. The results obtained in the present study are consistent with earlier findings. Rezvankhah et al. reported a 33.91% oil yield when applying MV to hempseed, alongside improvements in oxidative stability and antioxidant activity [23]. Similarly, Dal Pogetto et al. achieved a 30% oil yield after 60 min of microwave treatment when producing antioxidant-rich hempseed oil for incorporation into PBS/HSO active packaging films [30].

### 2.2. Fatty Acid Composition of Hempseed Samples: Influence of Extraction and Derivatisation Methods

The fatty acid composition of the investigated hempseed samples (Table 1) was strongly dominated by polyunsaturated fatty acids (PUFA), particularly linoleic acid (C18:2, ω-6) and α-linolenic acid (C18:3, ω-3), followed by monounsaturated fatty acids (MUFA), mainly oleic acid (C18:1, ω-9), and lower amounts of saturated fatty acids (SFA), such as palmitic (C16:0) and stearic acid (C18:0). Table 1 presents only the major fatty acids identified in the samples. In contrast, the complete fatty acid profile is provided in Supplementary Table S1. This compositional pattern is consistent with recent studies describing hempseed oil as a nutritionally valuable lipid source characterised by a high PUFA content and a balanced ω-6/ω-3 ratio, typically close to 3:1 [31,32,33].

**Figure 3:**
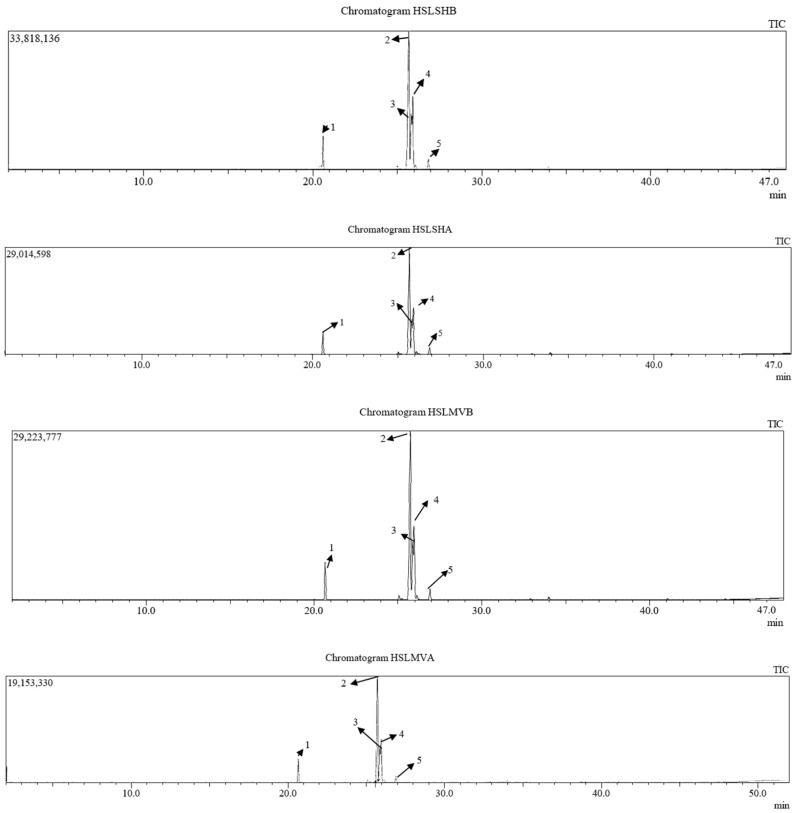
Representative GC–MS chromatograms of fatty acid methyl esters obtained from the same hempseed sample under the four extraction/derivatisation combinations used in this study. HSL—Lovrin 110; SHB—Soxhlet extraction followed by base-catalysed derivatisation; SHA—Soxhlet extraction followed by acid-catalysed derivatisation; MVB—microwave-assisted extraction followed by base-catalysed derivatisation; MVA—microwave-assisted extraction followed by acid-catalysed derivatisation. The numbered peaks correspond to the main FAMEs: 1, C16:0; 2, C18:2 ω-6; 3, C18:3 ω-3; 4, C18:1 ω-9; 5, C18:0. No co-eluting peaks interfering with the major FAMEs were observed under the chromatographic conditions employed.

To demonstrate the chromatographic separation performance achieved under the GC–MS conditions used in this study, representative chromatograms of the same hempseed sample analysed under the four extraction/derivatisation combinations are presented in Figure 3. The main FAME peaks were adequately resolved, supporting the semi-quantitative comparison of the fatty acid profiles obtained after Soxhlet and microwave-assisted extraction, each combined with either base- or acid-catalysed derivatisation.

Representative mass spectra, together with their NIST05 library matches for the two predominant FAMEs, methyl linoleate (C18:2 ω-6) and methyl oleate (C18:1 ω-9), are shown in Supplementary Figure S1, further confirming compound identification.

Statistical analysis revealed that the sample factor exerted a highly significant influence (*p* < 0.001) on all quantified fatty acids (Table 2), with partial eta squared values ranging from 0.794 to 0.993, underscoring the dominant contribution of biological variability to hempseed lipid composition. The three-way ANOVA further demonstrated that genotype differences markedly affected both major and minor fatty acids, reflecting the intrinsic heterogeneity of hempseed lipid profiles. This interpretation aligns with previous studies showing that genetic background and cultivation conditions modulate the relative proportions of linoleic, α-linolenic, and oleic acids, while the characteristic PUFA-rich profile of hempseed oil remains largely conserved across diverse germplasm [16,28,34]. The persistence of this PUFA-dominated pattern across all samples analysed in the present study reinforces the robustness and nutritional consistency of hempseed oil despite substantial biological variation.

The extraction method also significantly influenced fatty acid composition, with most fatty acids differing between Soxhlet and microwave-assisted extraction (*p* < 0.001, η^2^_p_ = 0.280–0.983). Pronounced effects were observed for C14:0, C16:1 (ω-7), C16:0, C18:1 (ω-9), C18:0, and long-chain saturated fatty acids (C20:0–C24:0), indicating that these fractions are particularly sensitive to extraction conditions. Microwave-assisted extraction yielded slightly higher proportions of SFA and MUFA, whereas Soxhlet extraction recovered higher PUFA levels, especially ω-3 fatty acids. These trends reflect the contrasting extraction mechanisms: Soxhlet enables exhaustive lipid recovery through prolonged solvent reflux, whereas microwave-assisted extraction relies on rapid dielectric heating, which can modify the extractability of thermolabile lipids. Similar observations have been reported in recent comparative studies [35]. The differences in fatty acid composition obtained by Soxhlet and microwave-assisted extraction should be interpreted with caution, as the extraction protocols differed not only in extraction principle but also in temperature, extraction time, and sample-to-solvent ratio, all of which may have influenced lipid recovery and the resulting fatty acid profiles. Notably, α-linolenic acid (C18:3, ω-3) did not differ significantly between extraction methods (*p* = 0.558), indicating high stability under the applied conditions. This agrees with recent findings showing that moderate microwave-assisted extraction preserves PUFA integrity in hempseed oil [36] and does not significantly alter linoleic or α-linolenic acid proportions compared with Soxhlet extraction [19]. Microwave pretreatment has also been associated with enhanced oxidative stability through the increased release of antioxidant compounds. A weaker but significant extraction effect was observed for linoleic acid (C18:2, ω-6) (*p* = 0.012), suggesting that major PUFAs are comparatively stable but still partially influenced by extraction parameters.

Derivatisation conditions further affected fatty acid quantification, with significant differences observed for C16:0, C18:1 (ω-9), C18:0, and long-chain saturated fatty acids (*p* < 0.001; η^2^_p_ = 0.449–0.992). In contrast, C20:4 (ω-3), C18:3 (ω-3), and C18:1 (ω-7) were not significantly affected. These results highlight the importance of derivatisation efficiency in FAME formation, as acid-catalysed methods generally ensure more complete conversion of complex lipid matrices. In contrast, base-catalysed methods may under-convert certain lipid fractions. Similar methodological effects have been reported in plant-derived matrices [37,38].

During our study, we identified pronounced interaction effects among the sample, extraction, and derivatisation factors, revealing an analytical complexity that has not been systematically documented for hempseed oil. The sample × extraction interaction was significant for several fatty acids, including C16:0, C18:1 (ω-9), and long-chain saturated fatty acids, demonstrating that extraction efficiency is not uniform across cultivars but depends on genotype-specific lipid characteristics. The sample × derivatisation interaction, significant for most fatty acids, further indicates that derivatisation performance is influenced by the underlying lipid matrix, highlighting the need to consider sample composition when selecting transesterification protocols. The extraction × derivatisation interaction—affecting C16:0, C20:4 (ω-3), C18:2 (ω-6), C18:1 (ω-9) and long-chain saturated fatty acids—indicates that these analytical steps exert combined rather than independent effects on fatty acid quantification. Most notably, the three-way interaction (sample × extraction × derivatisation) was significant for nearly all fatty acids, underscoring a complex interplay between biological variability and methodological parameters. This multifactor dependency provides new insight into how analytical workflows can shape the apparent lipid profile, an aspect often overlooked in routine fatty acid analysis. Comparable interaction patterns have been reported only recently in studies on plant-derived oils, which similarly emphasise that extraction methodology and processing conditions can substantially influence lipid composition [39,40].

At the level of individual fatty acids, linoleic acid (C18:2, ω-6) remained the dominant component across all samples, showing relatively stable values but still being significantly influenced by derivatisation and interaction effects. α-linolenic acid (C18:3, ω-3) exhibited greater variability between samples, suggesting a stronger dependence on biological factors. In contrast, oleic acid (C18:1, ω-9) was highly sensitive to all studied factors, indicating both biological and analytical influences. Long-chain saturated fatty acids (C20:0, C22:0, C24:0), although present in low concentrations, showed strong dependence on derivatisation and extraction conditions, highlighting their sensitivity to analytical procedures. This observation is consistent with recent reports indicating that minor fatty acids are more susceptible to methodological variability compared to major lipid components [31].

Our findings show that accurate fatty acid profiling requires consideration of genotype alongside extraction and derivatisation conditions, providing new methodological clarity for hempseed oil analysis. The results demonstrate that fatty acid composition is shaped by a multifactorial interplay between biological variability and analytical procedures, with strong interaction effects indicating that methodological steps cannot be evaluated independently. These insights highlight the need for greater standardisation in fatty acid analysis. In practical terms, Soxhlet extraction offers more comprehensive recovery of PUFA for nutritional characterisation, whereas microwave-assisted extraction provides faster processing and preferential recovery of more stable lipid fractions.

### 2.3. Lipid Quality Indices and Statistical Evaluation of Hempseed Samples

The lipid quality indices of the investigated hempseed samples (Table 3) confirm a highly favourable fatty acid profile, characterised by a clear predominance of polyunsaturated fatty acids (PUFA, ~66–75%) over saturated fatty acids (SFA, ~10–12%), resulting in elevated PUFA/SFA ratios (5.33–7.34). These values substantially exceed the recommended thresholds for cardiovascular protection and are consistent with recent studies that identify hempseed as a lipid source with significant functional potential [41,42]. In addition, the ω-6/ω-3 ratio ranged from 2.45 to 3.60, remaining within the optimal dietary range associated with reduced inflammatory and cardiovascular risk, consistent with recent compositional analyses of hempseed varieties [16].

The calculated atherogenic (AI) and thrombogenic (TI) indices were consistently low (AI: 0.078–0.096; TI: 0.096–0.141), while the hypocholesterolaemic/hypercholesterolaemic ratio (h/H) showed high values (10.3–12.7) (Table 3), indicating a lipid profile with reduced atherogenic and thrombogenic potential. These results are consistent with recent evaluations of plant lipid quality, which demonstrate that high PUFA levels and favourable fatty acid distribution directly improve lipid indices and confer cardioprotective effects [11,43].

The statistical analysis further supports these observations, as three-way ANOVA results (Table 4) revealed that the sample factor significantly influenced all lipid indices (*p* < 0.001), highlighting the dominant role of biological variability. This finding is consistent with recent studies demonstrating that genotype and cultivation conditions significantly affect fatty acid composition and derived nutritional indices in hempseed [16]. In addition, the extraction method and derivatization significantly affected most indices, confirming that analytical methodology substantially contributes to the variability in lipid quality parameters.

Method-dependent differences were evident when comparing extraction techniques (Table 3). Soxhlet extraction generally yielded higher PUFA, PUFA/SFA, and h/H values, suggesting a more exhaustive recovery of highly unsaturated fatty acids. In contrast, microwave extraction resulted in slightly higher SFA levels and lower unsaturation indices. These differences are reflected in the significant extraction effects identified in the three-way ANOVA (Table 4) and can be explained by the contrasting extraction mechanisms. Similar trends have been reported in recent studies, in which conventional solvent extraction preserved PUFA-rich profiles more effectively, whereas alternative techniques showed greater selectivity for more stable lipid fractions [44,45].

Importantly, the presence of significant interaction effects between sample, extraction, and derivatisation (Table 4) indicates that the influence of analytical conditions is matrix dependent. This suggests that lipid index responses are governed by both intrinsic compositional differences and methodological factors, reflecting the complexity of lipid extraction and analysis in plant-based systems. Similar interaction patterns have been increasingly reported in recent lipidomic studies [46,47], highlighting that extraction efficiency and lipid recovery are strongly dependent on both sample characteristics and analytical methodology, thereby emphasising the need for careful method selection.

The combined interpretation of compositional data (Table 3) and statistical analysis (Table 4) demonstrates that hempseed lipids exhibit a favourable fatty acid profile consistent with potential cardiovascular benefits, characterised by high PUFA content, optimal ω-6/ω-3 balance, low AI and TI values, and elevated h/H ratios. At the same time, the strong influence of methodological factors underscores the need for analytical standardisation to ensure reliable comparisons and accurate nutritional assessment.

### 2.4. Principal Component Analysis of Fatty Acid Profiles: Influence of Extraction and Derivatisation Methods

**Figure 4:**
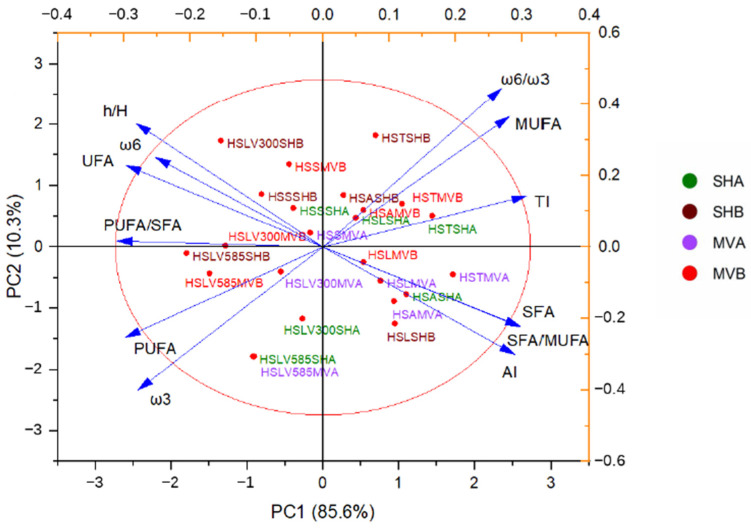
PCA biplot of hempseed samples based on fatty acid composition and lipid quality indices under different extraction and derivatisation conditions. Ellipses indicate 95% confidence intervals according to the extraction method. Hempseed sample: HSL—Lovrin 110, HSS—Silvana, HSA—Armanca, HST—Teodora, HSLV585—Lovrin 585, HSLV300—Lovrin 300. MV—microwave extraction; SH—Soxhlet extraction; A—acidic derivatisation; B—basic derivatisation.

Principal component analysis (PCA) was performed to assess the combined effects of extraction method (microwave, MV; Soxhlet, SH), derivatisation conditions (acidic, A; basic, B), and sample variability on the fatty acid composition of hempseed oil (Figure 4). The first two components explained 95.9% of the total variance, with PC1 accounting for 85.6% and PC2 for 10.3%, indicating an excellent representation of the dataset’s multivariate structure.

The PCA biplot shows a pronounced separation of samples along PC1, confirming that the extraction technique is the primary driver of compositional variability. MV-extracted samples cluster on the positive side of PC1, whereas SH-extracted samples are positioned toward the negative or near-zero region. The loading pattern reinforces this separation, with positive PC1 loadings for SFA (0.298), MUFA (0.281), SFA/MUFA (0.298), AI (0.289), TI (0.307), and the ω-6/ω-3 ratio (0.269), while negative PC1 loadings are associated with PUFA (−0.298), PUFA/SFA (−0.310), UFA (−0.297), ω-3 (−0.279), ω-6 (−0.253), and h/H (−0.282).

These patterns indicate that microwave extraction favours lipid fractions richer in SFA and MUFA, likely due to the greater thermal and oxidative stability of these fatty acids under rapid dielectric heating. This behaviour aligns with previous reports showing that microwave-assisted extraction can enhance the recovery of more stable lipid classes while altering PUFA distribution under specific processing conditions [48,49,50].

Conversely, the negative PC1 region, where SH samples cluster, is strongly associated with PUFA, PUFA/SFA, and ω-3 fatty acids. This reflects the exhaustive lipid recovery characteristic of Soxhlet extraction, which is known to better preserve and extract highly unsaturated fatty acids, including the nutritionally relevant ω-3 fraction [51]. The projection of SH samples toward PUFA-rich vectors is consistent with the intrinsic fatty acid profile of hempseed oil, dominated by linoleic (ω-6) and α-linolenic (ω-3) acids in a favourable ~3:1 ratio [52,53].

Although less pronounced, PC2 captures the secondary effects of derivatisation chemistry on fatty acid quantification. Acidic and basic derivatisation methods show partial separation along this axis. PC2 loadings reveal two contrasting patterns: positive loadings for ω-6/ω-3 (0.446), MUFA (0.365), and h/H (0.346), and negative loadings for ω-3 (−0.400), AI (−0.300), and SFA (−0.222); these patterns indicate opposing contributions of these variables to the secondary axis of variation.

These relationships suggest that derivatisation efficiency and selectivity influence the relative quantification of MUFA, long-chain SFA, and ω-3 fatty acids. Acid-catalysed transesterification typically ensures more complete conversion of complex lipid matrices, whereas base-catalysed methods may under-convert certain fractions, leading to subtle but measurable shifts in the resulting FAME profiles [54,55,56]. From a compositional perspective, the PCA reveals two major gradients in the lipid profiles: (i) a PUFA/ω-3 axis, associated with Soxhlet extraction, which has been widely reported in the literature as nutritionally important constituents of hempseed oil [41,57], and (ii) a MUFA/SFA axis, associated with microwave extraction. According to previous studies, these fatty acid classes differ in their susceptibility to oxidation, with PUFAs being generally more prone to oxidative degradation than MUFAs and SFAs [50].

Despite the inclusion of six hempseed samples, their intrinsic biological variability was less influential than the methodological factors. Samples clustered primarily according to extraction and derivatisation conditions, rather than origin, indicating that methodological effects partially obscure genotype in fatty acid composition. Nonetheless, the modest dispersion observed within clusters suggests that subtle sample-specific differences persist and may become more apparent under fully standardised analytical conditions.

The PCA demonstrates that the fatty acid composition of hempseed extracts is significantly modulated by analytical conditions, with the extraction method acting as the primary determinant and derivatisation as a secondary factor. The dominance of variables related to lipid quality indices (PUFA/SFA, AI, TI, h/H) further indicates that nutritional parameters play a central role in defining multivariate structure and sample differentiation. The clear separation between PUFA-rich and MUFA/SFA-rich profiles underscores the importance of method selection in lipid analysis, particularly when evaluating nutritional quality or comparing results across studies.

The present results indicate that no single analytical protocol is universally optimal for all lipidomic objectives. The choice of methodology should depend on the analytical endpoint. When rapid processing is the primary objective, microwave-assisted extraction may be the preferred option [19,23]. However, when comprehensive fatty acid profiling and nutritional lipid assessment are required, Soxhlet extraction appears to yield greater recovery of polyunsaturated fatty acids, particularly ω-3 fatty acids [23,58].

The combined PCA and ANOVA results demonstrate that both extraction and derivatisation significantly influence lipidomic characterisation, highlighting the importance of methodological standardisation for ensuring data reliability and inter-study comparability [59,60,61]. Therefore, for reference lipidomic characterisation of hempseed cultivars, Soxhlet extraction combined with a standardised and validated derivatisation protocol may represent the most suitable analytical approach when comprehensive fatty acid profiling and nutritional evaluation are the primary objectives [19,23,62,63].

### 2.5. Compositional Determinants of Oxidative Stability in Hempseed: Role of Fatty Acid Profile

**Figure 5:**
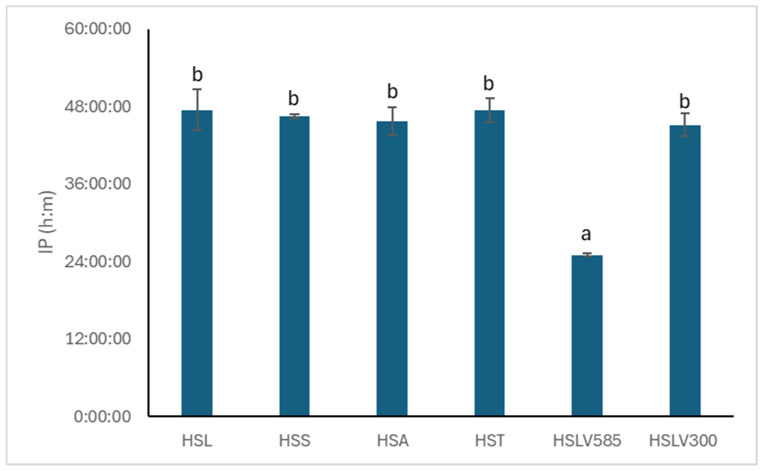
Oxidative stability of hempseed samples determined by Oxitest; hempseed sample: HSL—Lovrin 110, HSS—Silvana, HSA—Armanca, HST—Teodora, HSLV585—Lovrin 585, HSLV300—Lovrin 300, IP- Induction period. Values are expressed as mean ± standard deviation (SD) (*n* = 3). Different lowercase letters (a, b) indicate significant differences between samples according to the post hoc Tukey test (*p* < 0.05).

The oxidative stability of the hempseed samples, evaluated by the Oxitest method on ground seeds, showed noticeable differences between samples (Figure 5), with induction periods (IP) ranging from 25:00 to 47:29. The highest oxidative stability was observed for samples HSL (47:29) and HST (47:25), followed closely by HSS (46:26), HSA (45:42), and HSLV300 (45:07). At the same time, HSLV585 exhibited a markedly lower stability (25:00).

These results indicate that, although hemp seeds are characterised by a high content of polyunsaturated fatty acids (PUFAs), generally considered susceptible to oxidation, the oxidative behaviour of the analysed samples cannot be explained exclusively by the degree of unsaturation of the lipids. Hemp seeds also contain bioactive compounds with protective roles, such as tocopherols and phenolic compounds, which can significantly delay oxidative processes [52,64]. In this context, qualitative comparison with previously reported data on antioxidant activity for the same hemp varieties [65] highlights a coherent pattern: samples that show higher antioxidant activity also exhibit superior oxidative stability, while samples with reduced antioxidant activity are associated with shorter induction periods. Similar observations were also reported by Irakli et al., who demonstrated that differences in antioxidant activity between hemp genotypes directly influence oxidative stability [34].

Although the parameters were not determined in the same experimental setting, this convergence supports the hypothesis that intrinsic antioxidant systems play a key role in modulating lipid oxidation and compensating for the high level of unsaturation characteristic of hemp seeds. The relatively long induction periods observed for most samples suggest the presence of effective endogenous antioxidant systems, in particular tocopherols and phenolic compounds, known for their ability to delay lipid oxidation in oil matrices [53,66]. It is important to note that oxidative stability was measured on ground whole hemp seed samples, while fatty acid composition was analysed in extracted oil fractions. As a result, the observed oxidative behaviour reflects the influence of the entire seed matrix and cannot be attributed solely to the fatty acid profile. Recent studies confirm that the oxidative stability of hemp seeds and oil depends on the balance between the high PUFA content and the level of natural antioxidants, rather than exclusively on the fatty acid composition [16,41,42].

The markedly lower stability of sample HSLV585 may suggest either a higher susceptibility to oxidation or a lower content of antioxidant compounds compared to the other samples. This observation highlights the importance of sample-specific factors, such as cultivar differences, processing conditions, or storage history, which can significantly influence oxidative behaviour. Similar variability in oxidative stability among hempseed samples has been reported in the recent literature, where differences in antioxidant content and minor bioactive compounds were identified as key determinants of lipid stability [16]. Importantly, since oxidative stability was determined on ground hempseed samples, the results reflect the behaviour of the entire seed matrix, including lipids, proteins, and endogenous antioxidants, and are therefore independent of the extraction and derivatisation procedures used for fatty acid analysis. This makes Oxitest a valuable complementary tool to compositional analysis, providing functional insight into the product’s actual oxidative resistance.

The generally high oxidative stability observed across most samples is particularly relevant to the potential use of hempseed-derived materials in nutraceutical formulations and dietary supplements. The results suggest that the natural antioxidant systems present within the seed matrix may help maintain oxidative stability during storage. However, variability among samples indicates that careful selection of raw materials is essential to ensure stability and consistency in supplement formulations.

The oxidative stability of hempseed samples, expressed as induction period (IP), was influenced not only by fatty acid composition but also by the complex seed matrix (Table 5). Nevertheless, the negative correlation between PUFA content and IP may reflect the greater susceptibility of unsaturated bonds, particularly ω-3 fatty acids, to oxidative degradation, while the positive associations with SFA and MUFA may indicate that more saturated lipid fractions contribute to oxidative resistance.

However, unlike refined oils, the oxidative behaviour of whole hempseed cannot be attributed solely to fatty acid composition. Natural antioxidants such as tocopherols, phenolic compounds, and other bioactive constituents within the seed matrix serve a protective function, possibly reducing the pro-oxidative effects of high PUFA levels. This may explain why the correlations between IP and lipid fractions, although relatively strong, were not statistically significant (*p* > 0.05), indicating that additional factors beyond fatty acid composition contribute to oxidative stability.

The PUFA/SFA ratio also showed a negative association with IP (*r* = −0.713), confirming that increased unsaturation generally reduces oxidative stability. At the same time, the strong intercorrelations observed among lipid fractions (*r* > 0.97, *p* < 0.001) indicate that changes in fatty acid composition are structurally interdependent, reinforcing the role of overall lipid balance in determining oxidative behaviour.

The present findings further indicate that the oxidative behaviour of hempseed cannot be interpreted solely on the basis of fatty acid composition. Rather, oxidative stability appears to be influenced by the combined effects of lipid composition and matrix-associated antioxidant systems. Similar observations have been reported in recent studies, which emphasise that the oxidative stability of oilseeds is governed by both lipid composition and matrix-associated antioxidant systems [41,67].

Overall, the results demonstrate that oxidative stability in hempseed is a multifactorial property, primarily influenced by fatty acid unsaturation but significantly modulated by intrinsic seed components. This underscores the importance of considering the whole matrix when evaluating the stability and functionality of plant-based lipid sources.

## 3. Materials and Methods

### 3.1. Chemicals

The reagents, such as potassium hydroxide, sulfuric acid, and potassium hydrogen sulphate, were purchased from Sigma–Aldrich Chemie GmbH in Munich, Germany. Methanol and hexane were obtained from Merck KGaA in Darmstadt, Germany. All chemicals used for analysis were of analytical grade.

### 3.2. Plant Material

This investigation assessed a group of hemp (*Cannabis sativa* L.) seed genotypes, including the cultivars Lovrin 110—HSL, Silvana—HSS, Armanca—HSA, and Teodora—HST, along with two Lovrin breeding lines—LV 585—HSLV585 and LV 300—HSLV300, which are currently undergoing varietal certification. All plant material was grown under field conditions at the Lovrin Agricultural Research and Development Station (45°57′03″ N, 20°46′32″ E). All cultivars were grown under the same agronomic practices and environmental conditions. Representatives of mature seed lots from each genotype were used for analysis, reducing environmental variability among samples. Before laboratory analyses, seed lots were homogenised by grinding in a laboratory grinder (Grindomax GM200; Retsch GmbH, Düsseldorf, Germany) to ensure a uniform particle size and to enhance representativeness across tests.

**Figure 6:**
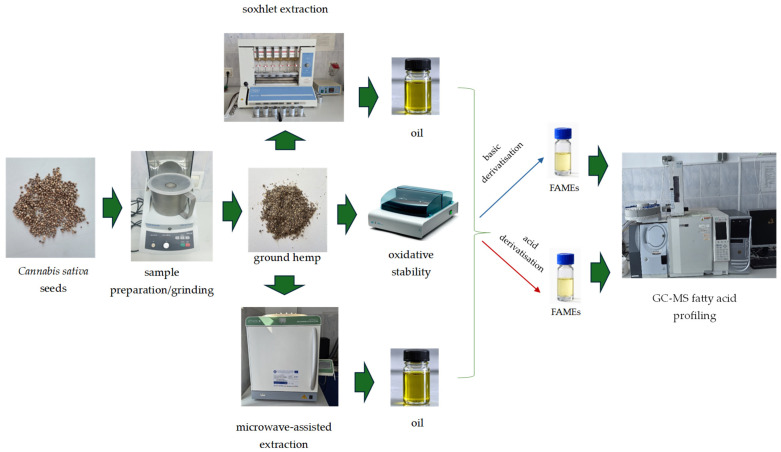
Schematic representation of the experimental workflow.

Figure 6 presents the graphical experimental workflow used in the study, which includes seed preparation, assessment of oxidative stability, oil extraction via Soxhlet or microwave-assisted method, and fatty acid profiling.

### 3.3. Soxhlet (SH) and Microwave-Assisted Extractions (MV)

Crude fat content was determined by Soxhlet extraction according to AOAC official method 920.39 [68] using hexane as the extraction solvent and a Soxtest Raypa SX-6 MP (Barcelona, Spain) apparatus operated at 94 °C for 180 min. Five grams of ground seeds were placed in the cartridges, with 50 mL of hexane used for each sample to extract the oil. The extracted oil was prepared in triplicate and stored in coloured glass vials at 4 °C.

The MV experiments used an ETHOS X (Advanced Microwave Extraction) system (Milestone, Sorisole, Italy). The extraction conditions were selected according to the standard extraction program provided by the instrument manufacturer. About 1 g of sample was weighed into the extraction vessel, and 20 mL of hexane was added. The vessels were sealed and heated with continuous stirring at a microwave power of 415 W, following this temperature schedule: heating to 100 °C for 10 min, then maintaining that temperature for 40 min. The extracted oil was prepared in triplicate and stored in coloured glass vials at 4 °C.

Oil yield (Y) for both the Soxhlet and MV was calculated as a percentage using the following formula:

(1) $$Y\;(\%)=\frac{M\;oil(g)}{M\;seed(g)}\times 100$$

where M oil—mass of oil extracted (in grams) and M seed—the mass of the seed sample used for extraction (in grams).

To compare the oil content obtained from the two extraction methods, SH and MV, the following indicator was used:

(2) $$Increase\;Oil\;MV/SH\;(\%)=[\frac{Oil\;MV-Oil\;SH}{Oil\;SH}]\times 100$$

where Oil MV—oil of the MV methods (g/100 g) and Oil SH—Oil of the SH methods (g/100 g).

### 3.4. Quantification of Fatty Acids Using Basic and Acid Derivatisation

Before GC analysis, fatty acids were converted to methyl esters (FAMEs) using two derivatisation methods. For the base-catalysed method, oils were dissolved in hexane (4 mL) and transesterified with 2 M KOH in methanol (400 µL), following Figueiredo et al. [69]. The mixture was vortexed for 5 min; potassium hydrogen sulphate (KHSO_4_, 500 mg) was added to quench and acidify; the phases were separated by centrifugation (3000 rpm, 15 min); and the hexane layer was transferred to GC vials.

For the acid-catalysed method, 0.5 g of the extracted oil was combined with hexane (20 mL) and the acid sulfuric–methanol mixture 6% (10 mL) and was then subjected to derivatisation in an ETHOS X (Advanced Microwave Extraction) system. The derivatisation conditions were applied according to the standard programme provided by the instrument manufacturer.

The vessels holding the samples were sealed and placed in the microwave oven. They were heated with continuous stirring according to the following temperature regimen: 85 °C for 3 min; at 4 min, the temperature reached 120 °C and was then sustained for 30 min. After cooling, the organic phase containing FAMEs was collected for GC analysis.

FAMEs were analysed using a Shimadzu GC–MS QP2010 Plus (Shimadzu, Tokyo, Japan) with MS detection and an AT-5MS capillary column (30 m × 0.32 mm i.d., 0.25 µm film). Samples (1.0 µL) were injected at 250 °C with helium as the carrier gas (1.8 mL min^−1^) under a 1:100 split. The oven programme began at 100 °C (2 min), increased at 3 °C min^−1^ to 150 °C (2 min hold), then at 5 °C min^−1^ to 250 °C, and finally at 10 °C min^−1^ to 300 °C (2 min hold). The mass spectrometer was operated in electron ionisation (EI) mode with a mass scan range of *m*/*z* 35–500. The MS parameters included an ion source temperature of 210 °C and an interface temperature of 255 °C. A certified Food Industry FAME Mix (Restek, Bellefonte, PA, USA) was analysed under the same chromatographic conditions to verify retention times and confirm the identification of fatty acid methyl esters. Identification of FAMEs was performed using the NIST05 spectral library, and quantification was performed using the area normalisation method. The results should be considered semi-quantitative and are expressed as the relative percentage of total FAMEs. Therefore, the data show the relative distribution of fatty acids within the lipid fraction, not their exact concentrations. As a result, variations due to extraction methods, derivatisation protocols, or genotypes should be interpreted with caution, since changes in specific fatty acids may reflect shifts in overall lipid composition rather than actual differences in their absolute levels. All analyses were performed in triplicate to ensure reproducibility.

### 3.5. Lipid Quality Indices

Lipid nutritional quality indices were calculated based on fatty acid composition, following the method described by Chen and Liu [2]. The specific indices analysed include

Atherogenic index (AI)

(3) $$AI=\frac{C12:0+(4\times C14:0)+C16:0}{\Sigma MUFA+\Sigma \omega \text{-}6+\Sigma \omega \text{-}3}$$

Thrombogenic index (TI)

(4) $$TI=\frac{C14:0+C16:0+C18:0}{0.5(\Sigma MUFA)+0.5(\Sigma \omega \text{-}6)+3(\Sigma \omega \text{-}3)+(\Sigma \omega \text{-}3/\Sigma \omega \text{-}6)}$$

Hypocholesterolaemic/hypercholesterolaemic ratio (h/H)

(5) $$h/H=\frac{cis\text{-}C18:1+\Sigma PUFA}{C12+C14:0+C16:0}$$

### 3.6. Oxidative Stability Determination Using OXITEST Velp

Oxidative stability was evaluated using the OXITEST reactor (VELP Scientifica, Usmate, (MB), Italy). Five grams of ground hempseed samples was weighed directly into the titanium oxidation chambers, avoiding preliminary lipid extraction. The test was performed under accelerated oxidation conditions at 90 °C and 6 bar. Oxygen consumption was monitored as a decrease in pressure in the sealed chambers, and the induction period (IP, h) was automatically determined from the oxidation curve by the instrument software. Results were expressed as mean ± standard deviation (SD) of triplicate determinations.

### 3.7. Statistical Analyses

All experimental analyses, including oil extraction, fatty acid determination, and oxidative stability measurements, were performed in triplicate (*n* = 3). Data are presented as mean ± standard deviation (SD), and statistical analyses were conducted using the values obtained from the three independent replicates. Statistical analyses were performed using JASP software (version 0.19.3.0, University of Amsterdam, Amsterdam, The Netherlands). Three-way ANOVA was used to evaluate the effects of sample, extraction method, derivatisation condition, and their interactions on fatty acid composition and lipid quality indices, followed by Pearson’s correlation analysis. Statistical significance was established at *p* < 0.05. In addition to *p*-values, partial eta squared (η^2^_p_) was calculated as a measure of effect size to assess the magnitude of the main and interaction effects. Multivariate analysis was performed by principal component analysis (PCA) using OriginPro 2025 software (OriginLab Corporation, Northampton, MA, USA). Variables were autoscaled prior to analysis, and PCA loadings and score plots were used to evaluate sample discrimination and variable contribution.

## 4. Conclusions

This study provides a comprehensive evaluation of the fatty acid composition, lipid quality indices, and oxidative stability of hempseed, demonstrating its strong potential as a nutraceutical raw material. Across all cultivars, hempseed lipids were characterised by high levels of polyunsaturated fatty acids—particularly linoleic and α-linolenic acids—resulting in favourable nutritional indices, including elevated PUFA/SFA and h/H ratios, low AI and TI values, and a balanced ω-6/ω-3 ratio. These attributes support the suitability of hempseed-derived lipids for cardiovascular and metabolic health applications.

Multivariate (PCA) and statistical (three-way ANOVA) analyses showed that the analytical methodology was a major source of variability, with both extraction and derivatisation significantly influencing the apparent fatty acid profile. The contrast between PUFA-rich profiles obtained by Soxhlet extraction and MUFA/SFA-enriched profiles obtained by microwave-assisted extraction underscores the need for standardised analytical protocols to ensure accurate characterisation and comparability of lipid-based nutraceuticals. The present results indicate that no single analytical protocol can be considered universally optimal for all lipidomic objectives. Under the experimental conditions evaluated in this study, microwave-assisted extraction resulted in higher extraction efficiency and shorter processing time, suggesting that it may be advantageous when rapid oil recovery is the main analytical or technological objective. However, for comprehensive fatty acid profiling and nutritional lipid assessment, Soxhlet extraction appeared more appropriate, as it favoured the recovery of polyunsaturated fatty acids, particularly ω-3 fatty acids. Consequently, for reference lipidomic characterisation of hempseed cultivars and inter-study comparisons, Soxhlet extraction combined with a standardised and validated derivatisation protocol may provide the most suitable analytical framework among the methodologies evaluated.

The oxidative stability assessment further revealed that lipid unsaturation alone does not fully explain oxidative behaviour in hempseed. Instead, the data indicate a matrix-dependent stabilisation mechanism, in which endogenous antioxidants—such as tocopherols and phenolic compounds—counterbalance the pro-oxidative effects of high PUFA levels. The alignment between oxidative stability and previously reported antioxidant activity for the same cultivars supports this interpretation and highlights the importance of considering both fatty acid composition and bioactive constituents when evaluating stability.

This study identifies hemp seeds as a valuable functional ingredient, combining high nutritional quality with notable oxidative stability. At the same time, the results highlight the need to control both biological variability and analytical methodology to obtain reliable lipid profiles. Standardisation of extraction and derivatisation procedures, together with the investigation of genotype effects, will be essential for advancing hemp seed lipidomics and supporting the development of hemp seed oils and derivatives for functional food and supplement applications.

## Figures and Tables

**Table 1:** Major fatty acid composition (% total fatty acids) of hempseed samples under different extraction methods and derivatisation conditions.

Sample	Extraction	Derivatisation	C16:0	C18:2 ω-6	C18:3 ω-3	C18:1 ω-9	C18:0
HSL	SH	B	8.056 ± 0.068	51.617 ± 0.228	16.129 ± 0.068	18.131 ± 0.015	3.045 ± 0.012
	SH	A	7.690 ± 0.032	52.932 ± 0.105	15.296 ± 0.012	18.171 ± 0.018	2.822 ± 0.014
	MV	B	7.817 ± 0.047	52.451 ± 0.155	15.713 ± 0.035	17.924 ± 0.022	2.833 ± 0.015
	MV	A	8.026 ± 0.043	52.944 ± 0.145	15.477 ± 0.030	17.372 ± 0.025	2.838 ± 0.017
HSS	SH	B	6.971 ± 0.074	53.844 ± 0.247	17.578 ± 0.076	15.710 ± 0.029	3.128 ± 0.018
	SH	A	7.189 ± 0.071	53.673 ± 0.235	16.938 ± 0.071	16.344 ± 0.032	3.158 ± 0.020
	MV	B	7.082 ± 0.084	53.908 ± 0.278	16.192 ± 0.090	16.835 ± 0.036	3.101 ± 0.021
	MV	A	7.403 ± 0.035	54.097 ± 0.117	16.618 ± 0.018	15.715 ± 0.039	3.107 ± 0.023
HAS	SH	B	7.543 ± 0.055	53.099 ± 0.184	15.389 ± 0.048	18.265 ± 0.043	2.996 ± 0.025
	SH	A	7.996 ± 0.032	52.577 ± 0.106	15.170 ± 0.013	17.919 ± 0.046	3.149 ± 0.026
	MV	B	7.695 ± 0.043	52.885 ± 0.144	14.943 ± 0.030	18.502 ± 0.050	2.993 ± 0.028
	MV	A	8.054 ± 0.060	52.835 ± 0.201	15.493 ± 0.055	17.305 ± 0.053	3.017 ± 0.029
HST	SH	B	7.629 ± 0.032	52.583 ± 0.105	13.963 ± 0.012	20.434 ± 0.057	2.945 ± 0.031
	SH	A	8.034 ± 0.042	52.033 ± 0.140	13.702 ± 0.028	20.353 ± 0.060	3.052 ± 0.032
	MV	B	7.879 ± 0.069	52.474 ± 0.230	14.127 ± 0.068	19.776 ± 0.064	2.964 ± 0.034
	MV	A	8.323 ± 0.063	52.168 ± 0.209	13.905 ± 0.059	19.425 ± 0.067	3.053 ± 0.035
HSLV 585	SH	B	6.928 ± 0.043	53.662 ± 0.144	20.197 ± 0.030	13.367 ± 0.071	2.576 ± 0.037
	SH	A	7.382 ± 0.065	53.012 ± 0.218	19.874 ± 0.063	13.200 ± 0.074	2.729 ± 0.039
	MV	B	7.096 ± 0.079	53.637 ± 0.262	19.603 ± 0.083	13.455 ± 0.078	2.519 ± 0.040
	MV	A	7.506 ± 0.030	53.341 ± 0.101	19.702 ± 0.011	12.914 ± 0.081	2.588 ± 0.042
HSLV 300	SH	B	6.930 ± 0.078	54.883 ± 0.261	17.478 ± 0.083	15.304 ± 0.085	2.631 ± 0.043
	SH	A	7.549 ± 0.072	53.341 ± 0.240	18.170 ± 0.073	14.577 ± 0.088	2.905 ± 0.045
	MV	B	7.178 ± 0.050	54.194 ± 0.168	19.080 ± 0.041	13.643 ± 0.092	2.667 ± 0.046
	MV	A	7.525 ± 0.039	54.332 ± 0.131	17.779 ± 0.024	14.314 ± 0.095	2.711 ± 0.048

Hempseed samples: HSL—Lovrin 110, HSS—Silvana, HSA—Armanca, HST—Teodora, HSLV585—Lovrin 585, HSLV300—Lovrin 300, MV—microwave extraction; SH—Soxhlet extraction; A—acidic derivatisation; B—basic derivatisation. Values are expressed as mean ± standard deviation (SD) (*n* = 3) as a percentage of total fatty acids.

**Table 2:** Effects of sample, extraction method, derivatisation, and their interactions on fatty acid composition (three-way ANOVA, *p*-values, partial eta squared, η^2^_p_).

Factor	C14:0	C16:1 ω-7	C16:0	C20:4 ω-3	C18:2 ω-6	C18:3 ω-3	C18:1 ω-9	C18:1 ω-7	C18:0	C20:1 ω-9	C20:0	C22:0	C24:0
Sample	<0.001 (0.794)	<0.001 (0.865)	<0.001 (0.982)	<0.001 (0.951)	<0.001 (0.937)	<0.001 (0.978)	<0.001 (0.993)	<0.001 (0.823)	<0.001 (0.981)	<0.001 (0.831)	<0.001 (0.829)	<0.001 (0.976)	<0.001 (0.956)
Extraction	<0.001 (0.473)	<0.001 (0.948)	<0.001 (0.694)	<0.001 (0.330)	0.012 (0.125)	0.558 (0.007)	<0.001 (0.541)	<0.001 (0.280)	<0.001 (0.614)	<0.001 (0.962)	<0.001 (0.651)	<0.001 (0.972)	<0.001 (0.983)
Derivatisation	<0.001 (0.467)	<0.001 (0.813)	<0.001 (0.924)	0.167 (0.039)	<0.001 (0.223)	0.116 (0.051)	<0.001 (0.449)	0.114 (0.051)	<0.001 (0.572)	<0.001 (0.967)	<0.001 (0.843)	<0.001 (0.989)	<0.001 (0.992)
Sample × Extraction	0.008 (0.268)	0.157 (0.144)	<0.001 (0.366)	0.766 (0.051)	0.156 (0.149)	<0.001 (0.346)	<0.001 (0.507)	0.003 (0.304)	<0.001 (0.376)	<0.001 (0.859)	<0.001 (0.720)	<0.001 (0.953)	<0.001 (0.940)
Sample × Derivatisation	0.095 (0.186)	<0.001 (0.421)	<0.001 (0.809)	0.006 (0.283)	<0.001 (0.696)	0.079 (0.181)	0.006 (0.280)	<0.001 (0.493)	<0.001 (0.732)	<0.001 (0.935)	<0.001 (0.782)	<0.001 (0.952)	<0.001 (0.943)
Extraction × Derivatisation	0.103 (0.058)	0.630 (0.004)	0.046 (0.080)	<0.001 (0.258)	<0.001 (0.273)	0.812 (0.001)	0.006 (0.145)	0.009 (0.134)	0.011 (0.127)	<0.001 (0.903)	<0.001 (0.656)	0.232 (0.025)	<0.001 (0.387)
Sample × Extraction × Derivatisation	0.008 (0.278)	<0.001 (0.448)	<0.001 (0.655)	0.053 (0.198)	<0.001 (0.518)	<0.001 (0.408)	<0.001 (0.594)	<0.001 (0.530)	<0.001 (0.636)	<0.001 (0.862)	<0.001 (0.503)	<0.001 (0.869)	<0.001 (0.926)

Statistical analysis was performed using three-way ANOVA (factors: sample, extraction method, derivatisation condition). Interaction effects were included. Significance was accepted at *p* < 0.05. Partial eta squared (η^2^_p_) was used as a measure of effect size.

**Table 3:** Nutritional and atherogenicity-related lipid indices of hempseed samples as affected by extraction technique and derivatisation method.

Sample	Extraction	Derivation	SFA	MUFA	PUFA	PUFA/ SFA	ω-3	ω-6	ω-6/ω-3	AI	TI	h/H
HSL	SH	B	12.090 ± 0.087	19.163 ± 0.031	68.747 ± 0.312	5.686 ± 0.015	17.129 ± 0.084	51.617 ± 0.228	3.013 ± 0.001	0.092 ± 0.0003	0.128 ± 0.0004	10.698 ± 0.055
	SH	A	11.589 ± 0.050	19.222 ± 0.035	69.189 ± 0.128	5.970 ± 0.015	16.257 ± 0.023	52.932 ± 0.105	3.256 ± 0.002	0.087 ± 0.0003	0.124 ± 0.0004	11.280 ± 0.029
	MV	B	11.710 ± 0.068	19.022 ± 0.042	69.269 ± 0.203	5.916 ± 0.017	16.819 ± 0.048	52.451 ± 0.155	3.119 ± 0.0004	0.089 ± 0.0004	0.124 ± 0.0004	11.052 ± 0.040
	MV	A	11.963 ± 0.067	18.566 ± 0.046	69.437 ± 0.188	5.804 ± 0.017	16.493 ± 0.043	52.944 ± 0.145	3.210 ± 0.0004	0.092 ± 0.0003	0.128 ± 0.0004	10.722 ± 0.033
HSS	SH	B	10.994 ± 0.102	16.641 ± 0.052	72.365 ± 0.340	6.582 ± 0.030	18.521 ± 0.093	53.844 ± 0.247	2.907 ± 0.001	0.079 ± 0.0006	0.111 ± 0.0006	12.549 ± 0.087
	SH	A	11.268 ± 0.099	17.228 ± 0.055	71.504 ± 0.322	6.345 ± 0.027	17.830 ± 0.087	53.673 ± 0.262	3.010 ± 0.002	0.081 ± 0.0005	0.116 ± 0.0006	12.132 ± 0.076
	MV	B	11.123 ± 0.116	17.761 ± 0.063	71.116 ± 0.386	6.394 ± 0.032	17.208 ± 0.108	53.908 ± 0.278	3.133 ± 0.004	0.080 ± 0.0006	0.116 ± 0.0005	12.317 ± 0.093
	MV	A	11.516 ± 0.064	16.773 ± 0.066	71.683 ± 0.147	6.224 ± 0.022	17.586 ± 0.030	54.097 ± 0.117	3.076 ± 0.001	0.084 ± 0.0002	0.119 ± 0.0005	11.713 ± 0.031
HAS	SH	B	11.455 ± 0.088	19.152 ± 0.072	69.392 ± 0.246	6.058 ± 0.025	16.293 ± 0.062	53.099 ± 0.184	3.259 ± 0.002	0.085 ± 0.0004	0.124 ± 0.0005	11.542 ± 0.048
	SH	A	12.359 ± 0.065	18.899 ± 0.078	68.741 ± 0.130	5.562 ± 0.019	16.165 ± 0.024	52.577 ± 0.106	3.253 ± 0.002	0.092 ± 0.0004	0.132 ± 0.0005	10.757 ± 0.021
	MV	B	11.622 ± 0.079	19.496 ± 0.083	68.881 ± 0.187	5.927 ± 0.024	15.997 ± 0.043	52.885 ± 0.144	3.306 ± 0.0001	0.087 ± 0.0003	0.127 ± 0.0005	11.250 ± 0.033
	MV	A	12.220 ± 0.099	18.415 ± 0.088	69.337 ± 0.271	5.674 ± 0.024	16.502 ± 0.070	52.835 ± 0.201	3.202 ± 0.001	0.092 ± 0.0002	0.130 ± 0.0005	10.668 ± 0.041
HST	SH	B	11.399 ± 0.070	21.347 ± 0.092	67.254 ± 0.128	5.900 ± 0.025	14.671 ± 0.023	52.583 ± 0.105	3.584 ± 0.002	0.086 ± 0.0002	0.131 ± 0.0006	11.431 ± 0.023
	SH	A	12.159 ± 0.083	21.349 ± 0.098	66.493 ± 0.181	5.469 ± 0.022	14.459 ± 0.041	52.033 ± 0.140	3.599 ± 0.0006	0.092 ± 0.0003	0.138 ± 0.0005	10.749 ± 0.026
	MV	B	11.896 ± 0.113	20.666 ± 0.102	67.437 ± 0.314	5.669 ± 0.028	14.963 ± 0.084	52.474 ± 0.230	3.507 ± 0.004	0.090 ± 0.0004	0.133 ± 0.0006	10.993 ± 0.051
	MV	A	12.539 ± 0.108	20.566 ± 0.107	66.863 ± 0.283	5.333 ± 0.024	14.695 ± 0.074	52.168 ± 0.209	3.550 ± 0.004	0.096 ± 0.0004	0.141 ± 0.0006	10.305 ± 0.037
HSLV585	SH	B	10.276 ± 0.089	14.338 ± 0.114	75.387 ± 0.187	7.337 ± 0.045	21.725 ± 0.043	53.662 ± 0.144	2.470 ± 0.002	0.078 ± 0.0002	0.096 ± 0.0006	12.623 ± 0.041
	SH	A	11.173 ± 0.113	14.224 ± 0.118	74.605 ± 0.297	6.678 ± 0.041	21.593 ± 0.079	53.012 ± 0.218	2.455 ± 0.001	0.084 ± 0.0004	0.103 ± 0.0006	11.703 ± 0.055
	MV	B	10.525 ± 0.130	14.530 ± 0.123	74.947 ± 0.362	7.121 ± 0.054	21.310 ± 0.100	53.637 ± 0.262	2.517 ± 0.0005	0.080 ± 0.0005	0.098 ± 0.0008	12.253 ± 0.078
	MV	A	11.129 ± 0.081	14.109 ± 0.128	74.728 ± 0.123	6.714 ± 0.038	21.387 ± 0.022	53.341 ± 0.240	2.494 ± 0.002	0.085 ± 0.0001	0.103 ± 0.0006	11.480 ± 0.017
HSLV300	SH	B	10.427 ± 0.131	16.258 ± 0.133	73.315 ± 0.361	7.031 ± 0.054	18.432 ± 0.100	54.883 ± 0.261	2.978 ± 0.002	0.078 ± 0.0005	0.105 ± 0.0008	12.689 ± 0.082
	SH	A	11.580 ± 0.128	15.750 ± 0.138	72.670 ± 0.330	6.275 ± 0.041	19.329 ± 0.090	53.341 ± 0.240	2.760 ± 0.0005	0.086 ± 0.0005	0.113 ± 0.0008	11.439 ± 0.056
	MV	B	10.656 ± 0.106	14.934 ± 0.145	74.409 ± 0.223	6.983 ± 0.049	20.216 ± 0.055	54.194 ± 0.168	2.681 ± 0.001	0.081 ± 0.0003	0.103 ± 0.0007	12.180 ± 0.041
	MV	A	11.257 ± 0.096	15.496 ± 0.149	73.211 ± 0.167	6.504 ± 0.040	18.879 ± 0.036	54.332 ± 0.131	2.878 ± 0.001	0.085 ± 0.0002	0.112 ± 0.0007	11.516 ± 0.023

Hempseed sample: HSL—Lovrin 110, HSS—Silvana, HSA—Armanca, HST—Teodora, HSLV585—Lovrin 585, HSLV300—Lovrin 300, SFA—saturated fatty acids; MUFA—monounsaturated fatty acids; PUFA—polyunsaturated fatty acids; UFA—unsaturated fatty acids. AI—atherogenic index; TI—thrombogenic index; h/H—hypocholesterolaemic/hypercholesterolaemic fatty acid ratio. MV—microwave extraction; SH—Soxhlet extraction; A—acidic derivatisation; B—basic derivatisation. Data are presented as mean ± standard deviation (SD) (*n* = 3).

**Table 4:** Three-way ANOVA results (*p*-values, partial eta squared, η^2^_p_) for lipid indices of hempseed samples under different extraction and derivatisation conditions.

Factor	SFA	MUFA	PUFA	PUFA/SFA	ω-3	ω-6	ω-6/ω-3	AI	TI	h/H
Sample	<0.001 (0.976)	<0.001 (0.992)	<0.001 (0.986)	<0.001 (0.995)	<0.001 (0.974)	<0.001 (0.937)	<0.001 (0.976)	<0.001 (0.995)	<0.001 (0.993)	<0.001 (0.994)
Extraction	<0.001 (0.342)	<0.001 (0.382)	0.068 (0.068)	<0.001 (0.280)	0.715 (0.003)	0.012 (0.125)	0.990 (<0.001)	<0.001 (0.901)	0.002 (0.182)	<0.001 (0.902)
Derivatisation	<0.001 (0.927)	<0.001 (0.313)	0.002 (0.181)	<0.001 (0.954)	0.289 (0.023)	<0.001 (0.223)	0.437 (0.013)	<0.001 (0.982)	<0.001 (0.869)	<0.001 (0.979)
Sample × Extraction	<0.001 (0.549)	<0.001 (0.491)	0.012 (0.256)	<0.001 (0.651)	0.010 (0.264)	0.156 (0.149)	<0.001 (0.341)	<0.001 (0.726)	<0.001 (0.480)	<0.001 (0.624)
Sample × Derivatisation	<0.001 (0.832)	0.011 (0.260)	0.004 (0.294)	<0.001 (0.904)	0.085 (0.178)	<0.001 (0.696)	<0.001 (0.375)	<0.001 (0.949)	<0.001 (0.651)	<0.001 (0.942)
Extraction × Derivatisation	0.225 (0.030)	0.015 (0.118)	0.076 (0.064)	<0.001 (0.210)	0.606 (0.006)	<0.001 (0.273)	0.399 (0.015)	<0.001 (0.223)	0.528 (0.008)	0.264 (0.026)
Sample × Extraction × Derivatisation	<0.001 (0.640)	<0.001 (0.517)	0.031 (0.220)	<0.001 (0.700)	<0.001 (0.355)	<0.001 (0.518)	<0.001 (0.463)	<0.001 (0.895)	<0.001 (0.513)	<0.001 (0.871)

Values represent *p*-values obtained from three-way ANOVA evaluating the effects of sample, extraction method, derivatization, and their interactions. Significant differences were considered at *p* < 0.05. Partial eta squared (η^2^_p_) was used as a measure of effect size. SFA—saturated fatty acids; MUFA—monounsaturated fatty acids; PUFA—polyunsaturated fatty acids. AI—atherogenic index; TI—thrombogenic index; h/H—hypocholesterolaemic/hypercholesterolaemic ratio.

**Table 5:** Pearson’s correlation coefficient between oxidative stability (IP) and fatty acid composition of hempseed samples.

Variable		IP	SFA		MUFA		PUFA		PUFA/SFA
1. IP	Pearson’s r	—							
	*p*-value	—							
2. SFA	Pearson’s r	0.676	—						
	*p*-value	0.141	—						
3. MUFA	Pearson’s r	0.720	0.970	**	—				
	*p*-value	0.106	0.001		—				
4. PUFA	Pearson’s r	−0.716	−0.980	***	−0.999	***	—		
	*p*-value	0.110	<0.001		<0.001		—		
5. PUFA/SFA	Pearson’s r	−0.713	−0.995	***	−0.988	***	0.994	***	—
	*p*-value	0.112	<0.001		<0.001		<0.001		—

** *p* < 0.01, *** *p* < 0.001.

## Data Availability

The original contributions presented in the study are included in the article. Further inquiries can be directed to the corresponding author.

## Supplementary Materials

The following supporting information can be downloaded at: https://www.mdpi.com/article/10.3390/molecules31142480/s1, Table S1: Complete fatty acid composition (% total fatty acids) of hempseed samples under different extraction and derivatisation conditions; Figure S1: Representative GC–MS mass spectra and NIST05 library matching for the identification of the major fatty acid methyl esters (FAMEs).
