# Supplementary material for: Method-Dependent Variability in Hempseed Lipidomics: Combined Influence of Extraction, Derivatisation, and Genotype on Fatty Acid Composition and Stability
Source: Molecules. 2026 Jul 16;31(14):2480. doi: 10.3390/molecules31142480 (PMC13414230; doi:10.3390/molecules31142480)
Supplement: Supplementary file 1 [file molecules-31-02480-s001.zip › molecules-4397905-supplementary.pdf]

Table S1. Complete fatty acid composition (% total fatty acids) of hempseed samples under different extraction and derivatisation conditions

Sample	Extraction	Derivatisation.	C14:0	C16:1 ω-7	C16:0	C20:4 ω-3	C18:2 ω-6	C18:3 ω-3	C18:1 ω-9	C18:1 ω-7	C18:0	C20:1 ω-9	C20:0	C22:0	C23:0	C24:0
**HSL**	SH	B	0.035±0.003	0.104±0.001	8.056±0.068	1.000±0.016	51.617±0.228	16.129±0.068	18.131±0.015	0.677±0.012	3.045±0.012	0.251±0.003	0.633±0.001	0.231±0.002	nd	0.091±0.001
	SH	A	0.032±0.001	0.107±0.001	7.690±0.032	0.962±0.011	52.932±0.105	15.296±0.012	18.171±0.018	0.707±0.013	2.822±0.014	0.237±0.003	0.725±0.001	0.219±0.001	nd	0.101±0.001
	MV	B	0.036±0.002	0.119±0.001	7.817±0.047	1.106±0.013	52.451±0.155	15.713±0.035	17.924±0.022	0.702±0.015	2.833±0.015	0.276±0.003	0.644±0.001	0.262±0.001	nd	0.117±0.001
	MV	A	0.044±0.002	0.133±0.002	8.026±0.043	1.016±0.016	52.944±0.145	15.477±0.030	17.372±0.025	0.732±0.016	2.838±0.017	0.329±0.003	0.611±0.001	0.295±0.003	0.033±0.002	0.149±0.001
**HSS**	SH	B	0.021±0.004	0.091±0.002	6.971±0.074	0.943±0.017	53.844±0.247	17.578±0.076	15.710±0.029	0.622±0.018	3.128±0.018	0.219±0.003	0.623±0.002	0.177±0.003	nd	0.073±0.001
	SH	A	0.027±0.004	0.091±0.002	7.189±0.071	0.893±0.016	53.673±0.235	16.938±0.071	16.344±0.032	0.591±0.019	3.158±0.020	0.203±0.002	0.605±0.002	0.202±0.001	nd	0.087±0.001
	MV	B	0.024±0.004	0.115±0.002	7.082±0.084	1.016±0.018	53.908±0.278	16.192±0.090	16.835±0.036	0.581±0.021	3.101±0.021	0.230±0.004	0.634±0.002	0.191±0.003	nd	0.091±0.002
	MV	A	0.029±0.001	0.121±0.002	7.403±0.035	0.968±0.012	54.097±0.117	16.618±0.018	15.715±0.039	0.626±0.022	3.107±0.023	0.312±0.003	0.593±0.002	0.269±0.002	0.027±0.002	0.116±0.002
**HAS**	SH	B	0.025±0.003	0.087±0.002	7.543±0.055	0.904±0.014	53.099±0.184	15.389±0.048	18.265±0.043	0.602±0.024	2.996±0.025	0.198±0.002	0.595±0.002	0.224±0.002	nd	0.071±0.002
	SH	A	0.031±0.001	0.097±0.003	7.996±0.032	0.995±0.011	52.577±0.106	15.170±0.013	17.919±0.046	0.671±0.026	3.149±0.026	0.212±0.004	0.736±0.002	0.324±0.002	nd	0.124±0.002
	MV	B	0.034±0.002	0.103±0.003	7.695±0.043	1.054±0.013	52.885±0.144	14.943±0.030	18.502±0.050	0.621±0.027	2.993±0.028	0.270±0.003	0.572±0.002	0.230±0.003	nd	0.098±0.002
	MV	A	0.034±0.003	0.125±0.003	8.054±0.060	1.009±0.015	52.835±0.201	15.493±0.055	17.305±0.053	0.643±0.028	3.017±0.029	0.342±0.004	0.655±0.002	0.328±0.003	0.029±0.001	0.132±0.002
**HST**	SH	B	0.034±0.001	0.070±0.003	7.629±0.032	0.708±0.011	52.583±0.105	13.963±0.012	20.434±0.057	0.620±0.030	2.945±0.031	0.223±0.002	0.552±0.002	0.159±0.002	nd	0.079±0.002
	SH	A	0.034±0.002	0.094±0.003	8.034±0.042	0.757±0.013	52.033±0.140	13.702±0.028	20.353±0.060	0.637±0.031	3.052±0.032	0.265±0.003	0.680±0.002	0.254±0.002	nd	0.104±0.002
	MV	B	0.036±0.003	0.103±0.003	7.879±0.069	0.836±0.016	52.474±0.230	14.127±0.068	19.776±0.064	0.636±0.033	2.964±0.034	0.152±0.001	0.627±0.002	0.273±0.002	nd	0.116±0.002

	MV	A	0.039±0.003	0.112±0.004	8.323±0.063	0.790±0.015	52.168±0.209	13.905±0.059	19.425±0.067	0.676±0.035	3.053±0.035	0.354±0.002	0.626±0.003	0.335±0.002	0.032±0.002	0.161±0.002
**HSLV 585**	SH	B	0.034±0.003	0.078±0.006	6.928±0.043	1.528±0.013	53.662±0.144	20.197±0.030	13.367±0.071	0.659±0.036	2.576±0.037	0.234±0.003	0.478±0.003	0.196±0.002	nd	0.063±0.001
	SH	A	0.035±0.003	0.096±0.004	7.382±0.065	1.719±0.016	53.012±0.218	19.874±0.063	13.200±0.074	0.717±0.038	2.729±0.039	0.211±0.002	0.651±0.003	0.253±0.001	nd	0.123±0.001
	MV	B	0.037±0.004	0.110±0.004	7.096±0.079	1.707±0.017	53.637±0.262	19.603±0.083	13.455±0.078	0.707±0.039	2.519±0.040	0.258±0.002	0.550±0.003	0.223±0.002	nd	0.100±0.001
	MV	A	0.047±0.001	0.117±0.004	7.506±0.030	1.685±0.011	53.341±0.101	19.702±0.011	12.914±0.081	0.752±0.040	2.588±0.042	0.327±0.002	0.602±0.003	0.258±0.003	0.033±0.003	0.130±0.002
**HSLV 300**	SH	B	0.032±0.003	0.092±0.003	6.930±0.078	0.954±0.017	54.883±0.261	17.478±0.083	15.304±0.085	0.678±0.042	2.631±0.043	0.183±0.002	0.600±0.001	0.170±0.002	nd	0.064±0.002
	SH	A	0.042±0.004	0.109±0.005	7.549±0.072	1.159±0.017	53.341±0.240	18.170±0.073	14.577±0.088	0.736±0.043	2.905±0.045	0.329±0.002	0.672±0.002	0.272±0.003	nd	0.141±0.002
	MV	B	0.034±0.002	0.117±0.005	7.178±0.050	1.136±0.014	54.194±0.168	19.080±0.041	13.643±0.092	0.928±0.045	2.667±0.046	0.246±0.003	0.487±0.002	0.202±0.002	nd	0.088±0.002
	MV	A	0.041±0.002	0.131±0.005	7.525±0.039	1.100±0.012	54.332±0.131	17.779±0.024	14.314±0.095	0.698±0.047	2.711±0.048	0.352±0.002	0.569±0.003	0.283±0.001	0.036±0.002	0.129±0.002

hempseed sample: HSL - Lovrin 110, HSS - Silvana, HSA - Armanca, HST - Teodora, HSLV585 – Lovrin 585, HSLV300 – Lovrin 300, MV – microwave extraction; SH – Soxhlet extraction; A – acidic derivatisation; B – basic derivatisation; nd -not detected. Values are expressed as mean ± standard deviation (SD) *(n* = 3) as a percentage of total fatty acids

(A)

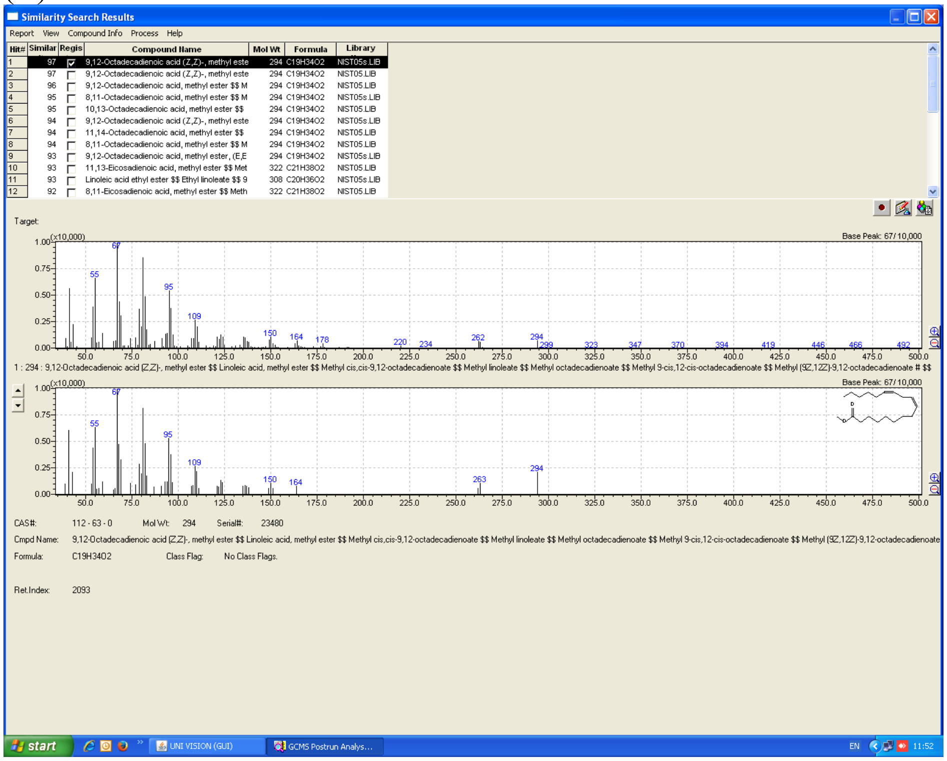

(B)

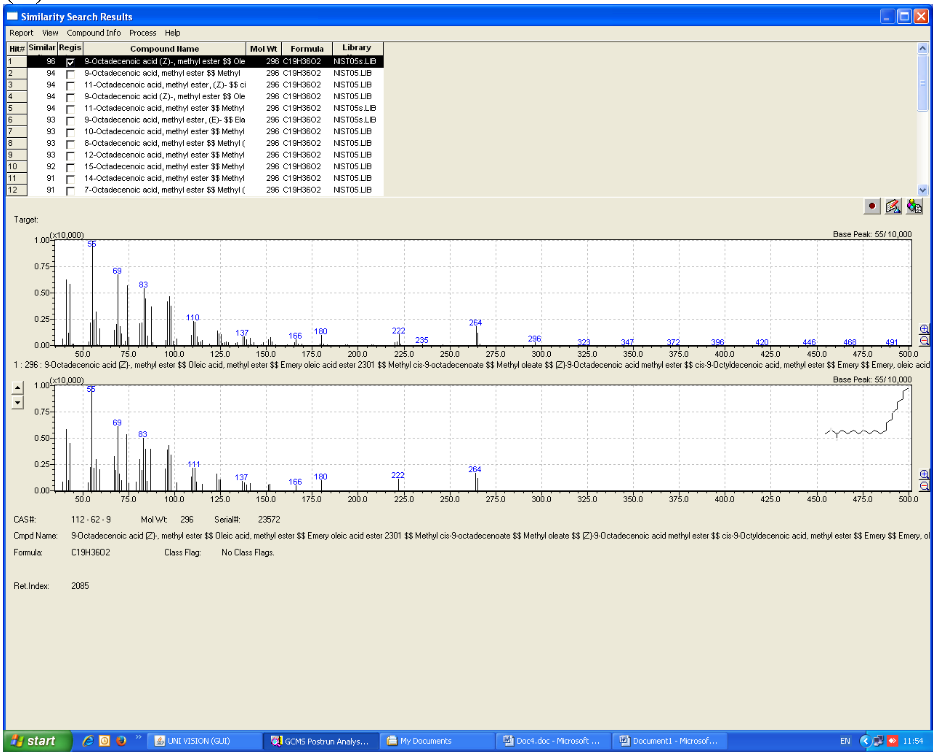

Figure S1. Representative GC–MS mass spectra and NIST05 library matching for the identification of the major fatty acid methyl esters (FAMEs): (A) methyl linoleate (C18:2 ω-6) and (B) methyl oleate (C18:1 ω-9).
